# Transcriptome Analysis of Purple Pericarps in Common Wheat (*Triticum aestivum* L.)

**DOI:** 10.1371/journal.pone.0155428

**Published:** 2016-05-12

**Authors:** Di Liu, Shiming Li, Wenjie Chen, Bo Zhang, Dengcai Liu, Baolong Liu, Huaigang Zhang

**Affiliations:** 1 Key Laboratory of Adaptation and Evolution of Plateau Biota (AEPB), Northwest Institute of Plateau Biology, Chinese Academy of Sciences, Qinghai, Xining, 810008, China; 2 Qinghai Province Key Laboratory of Crop Molecular Breeding, Xining, 810008, China; Murdoch University, AUSTRALIA

## Abstract

Wheat (*Triticum aestivum* L.) cultivars possessing purple grain arethought to be more nutritious because of high anthocyanin contents in the pericarp. Comparative transcriptome analysis of purple (cv Gy115) and white pericarps was carried out using next-generation sequencing technology. There were 23,642 unigenes significantly differentially expressed in the purple and white pericarps, including 9945 up-regulated and 13,697 down-regulated. The differentially expressed unigenes were mainly involved in encoding components of metabolic pathways, The flavonoid biosynthesis pathway was the most represented in metabolic pathways. In the transcriptome of purple pericarp in Gy115, most structural and regulatory genes biosynthesizing anthocyanin were identified, and had higher expression levels than in white pericarp. The largestunigene of anthocyanin biosynthesis in Gy115 was longer than the reference genes, which implies that high-throughput sequencing could isolate the genes of anthocyanin biosynthesis in tissues or organs with high anthocyanin content. Based on present and previous results, three unigenes of *MYB* gene on chromosome 7BL and three unigenes of *MYC* on chromosome 2AL were predicted as candidate genes for the purple grain trait. This article was the first to provide a systematic overview comparing the transcriptomes of purple and white pericarps in common wheat, which should be very valuable for identifying the key genes for the purple pericarp trait.

## Introduction

White- and red-grained bread wheat (*Triticum aestivum* L.) types are common, while few wheat cultivars possess either purple or blue grain. Purple and blue grains are thought to result from accumulation of anthocyanin in pericarp or aleurone[1–2]. Because of the high anthocyanin content, this kind of wheat is thought to be more nourishing than red or white wheat[3]. The purple or blue grain could also be used as a marker for special purpose wheat to distinguish them from others, for instance in an indirect selection program to measure the degree of natural cross pollination and of gamete transmission, histological observations and pigment characterization [4].

Anthocyanins are water-soluble pigments endemic to plants, and can endow plant organs and tissues with a red, purple or blue color [5]. The traits related to anthocyanin biosynthesis can be easily observed. Moreover, as a type of secondary metabolite, less anthocyanin has almost no effect on plant growth and development, and so no lethality occurs. These special characteristics make the anthocyanin biosynthesis pathway clearer than other metabolic pathways in model plants [6]. Anthocyanin biosynthesis is first induced by phenylalanine after three steps of enzyme promoting reaction ofone molecule of p-coumaricacyl CoA and three molecules of propylene acyl CoA in the presence of chalcone synthase (CHS) generating chalcone; however, little chalcone accumulates due to rapid isomerization and the formation of naringenin (flavanone) with the catalysis of chalcone isomerase (CHI). Flavanone to flavonoid-3-hydroxylase (F3H) is catalyzed by C-ring position-3 hydroxylation to form two flavanonols. These two flavanonols are the substrates for the two enzymes flavonoid-3'-hydroxylase (F3ʹH) and B ring hydroxylase F3ʹ5ʹH (flavonoid-3ʹ,5ʹ-hydroxylase) substrate. F3H, F3ʹH and F3ʹ5ʹH belong to the P450 super family. The products of catalytic reaction of three hydroxylase F3H, F3ʹH and F3ʹ5ʹH are direct precursors in the synthesis of anthocyanins. The reaction from two hydrogen to anthocyanin is very complex, and requires a number of different enzymes. Anthocyanidin reductase (ANS) is a double-plus oxygen enzyme that catalyzes the transformation from a colorless to a colored flower[6–8]. Then, different glycosylation genes begin to work, and generate many kinds of anthocyanins. Generally, the anthocyanin biosynthesis pathway is regulated by MYB, bHLH and WD40 proteins [8,9]. The anthocyanin biosynthesis pathway belongs to the phenylalanine pathway, and results in biosynthesis of many compounds, such as proanthocyanidin, catechin, epicatechin, flavonols and flava-4-ol [6,10,11]. Some structural genes compete for the same substrates as the structural genes of anthocyanin biosynthesis to produce these different compounds.

Wheat cultivars with colored grains have been planted for several decades, and attempts to determine the cause of the phenotypes have been made since the phenotypes were recognized. Hexaploid wheat with blue grains is thought to be the result of the artificial introgression of genes from *Agropyron* species [3], while purple grains occur in tetraploid wheat from Ethiopia and in one bread wheat accession apparently native to China, according to the literature [12,13]. Classic genetic research showed that the key gene for blue grains was on chromosome 4E of *Agropyron* species, while purple grain was respectively controlled by two complementary genes *Pp-1* and *Pp-3* [14,15,16]. *Pp-3*is located on chromosome 2AL, and *Pp-1* has three homoalleles on each of the group 7 chromosomes [15]. However, the exact genes have not been isolated and verified. Currently, high-throughput RNA sequencing (RNA-Seq) has emerged as a powerful and cost-efficient tool for transcriptome analysis and transcript profiling in various plant species[17–19]. RNA-Seq has a large advantage in providing information on the nucleotide sequence of genes expressed in the transcriptome.In this article, RNA-Seqwas used to determine the transcriptome difference between purple and white pericarps, and isolate the dominant genes for the purple grain trait.

## Materials and Methods

### Plant materials and RNA extraction

Common wheat (*T*. *aestivum* L., 2n = 6x = 42, AABBDD) cultivars Opata and Gaoyuan115 (Gy115) were used in this paper. Seeds of Opata were obtained from the International Triticeae Mapping Initiative (ITMI). Gy115 is a commercial cultivar, developed by the Northwest Institute of Plateau Biology, Chinese Academy of Sciences, Xining, Qinghai, China. The purple grain trait of Gy115 was from tetraploid wheat cultivar Kugua. At 14 d after flowering, the pericarp was carefully stripped from each grain, and they were immediately placed in liquid nitrogen. The pericarp of 10 grains per cultivar was collected for total RNA extraction.

Total RNA was extracted using the Tiangen RNAprep Pure Plant Kit (Tiangen, China) according to the standard protocol. The quality of total RNA was checked by electrophoresis in a 1.0% agarose gel and the concentration of total RNA was determined by NanoDrop (Thermo Scientific, Wilmington, DE, USA).S1 Fig shows a total RNA gel-like image produced by the Bioanalyzer (S1 Fig). The RNA concentration of Opata and GY115 is 836ng/ul, 567ng/ul respectively.

### cDNA preparation and Illumina sequencing

The two cDNA libraries of pericarps of Opata and GY115 were prepared according to the manufacturer’s instructions for mRNA-Seq sample preparation (Illumina Inc, San Diego, CA, USA). The cDNA library products were sequenced by Illumina paired-end sequencing technology with read lengths of 100bp, and they were sequenced on the Illumina HiSeq 2000 instrument by Huada Technologies Co., Ltd. (Beijing, China).They were sequenced three times on technical replicates. The dataset was submitted to the NIH Short Read Archive (accession number: SRP068397). It is public in SRA Browser: http://trace.ncbi.nlm.nih.gov/Traces/study/?acc=SRP068397+&go=go.

### Sequence data filtering and de novo assembly

Before assembly, the raw paired-end reads produced from sequencing machines were filtered to obtain high-quality clean reads. Low quality sequences were removed, including sequences with ambiguous bases (denoted with > 5% ‘N’ in the sequence trace), low quality reads (the rate of reads with quality value ≤ 10 was more than 20%) and reads with adaptors. After purity filtering was completed, the high-quality reads were assembled by Trinity with default parameters to construct unique consensus sequences [20].

All these unigene sequences were compared to protein databases using blastx(e-value<0.00001)in the following order: Nr, Swiss-Prot, KEGG and COG. Then the blast results were used to extract CDS from unigene sequences and to translate them into peptide sequences. The blast results were also used to train ESTScan [21].

### Analysis of differential gene expression

The unigene expression levels were calculated by the FPKM values (fragments per kb per million reads). Unigenes that were differentially expressed between the Opata and GY115 were analyzed by Chi-square test using IDEG6 software [22]. The false discovery rate (FDR) method was introduced to determine the threshold p-value at FDR ≤ 0.001; and the absolute value of |log2Ratio| ≥ 1 was used as the threshold to determine the significance of the differential expression of unigenes.

### Gene annotation and classification

In order to perform functional annotation, the assembled unigenes were submitted to a public database and compared with the NCBI non-redundant protein database (NR), Swiss-Prot (http://www.uniprot.org/), Clusters of Orthologous Groups (COG) (http://www.ncbi.nlm.nih.gov/COG/) and Kyoto Encyclopedia of Genes and Genomes (KEGG) databases (http://www.genome.jp/kegg/) using blastx(v.2.2.26) with e-value < 1e-5, while the Gene Ontology (GO) annotations were analyzed using the Blast2GO(V.2.5) program (http://www.geneontology.org). All differentially abundant unigenes between purple and white pericarps of cultivars Gy115 and Opata were mapped to the GO and KEGG pathway databases, and then the respective numbers of unigenes for every GO and KEGG orthology (KO) term were calculated. To compare these unigenes with the whole transcriptome background from wheat, significantly enriched GO and KO terms from the set of differentially abundant unigenes were identified using the hypergeometric test [23].

### Location of unigenes in the wheat genome

The genes related to anthocyanin biosynthesis in KEGG pathways were collected and aligned to the unigenes of the pericarp transcriptome in Gy115 and the mixture of pericarp transcriptomes of Gy115 and Opata using blastx software with e-value < 1e-5, respectively. The length of these unigenes of anthocyanin biosynthesis in the pericarp transcriptomeofGy115 were compared with reference genes to evaluate the possibility of isolating the genes related to anthocyanin synthesis in tissues or organs with high anthocyanin contentsusing next-generation sequencing technology.

The FPKM values of unigenes aligned to genes of the anthocyanin biosynthesis pathway were accumulated together to compare their relative expression levels. The unigenes related to anthocyanin biosynthesis were aligned to chromosomes based on blastx software with e-value < 1e-5 and a reference database (ta_IWGSC_MIPSv2.2_HighConf_CDS_2014Jul18.fa).

## Results

### Transcriptome analyses of two wheat cultivars

**Fig 1:**
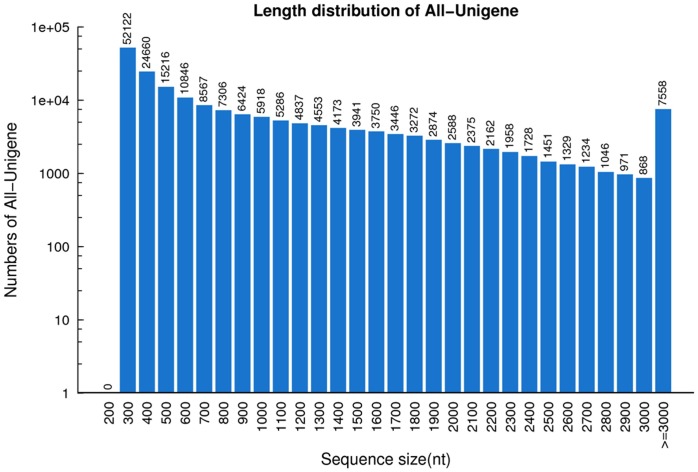
Distribution of lengths of the assembled unigenes in wheat pericarps.

After filtering, a total of 97,155,472 reads for Opata and 89,469,748 for GY115 remained, with a Q20 percentage of 97.88%. The high-quality reads were aligned to assemble unigenes (Table 1). Trinity [20] software was used to generate 735,638 contigs, which were further assembled into 192,459 unigenes with an average length of 922 nt and an N50 length of 1580 nt(Fig 1). A total of 103,790 CDSs were detected from these unigenes (Table 2).

**Table 1:** Summary of transcriptome sequencing data in the GY115 and Opata.

	GY115	Opata
**Total Raw Reads**	97,020,510	105,387,672
**Total Clean Reads**	89,469,748	97,155,472
**Total Clean Nucleotides (nt)**	8,052,277,320	8,743,992,480
**Q20 percentage**	97.88%	97.88%
**N percentage**	0.00%	0.00%
**GC percentage**	52.79%	52.35%

**Table 2:** Statistics for CDS predictions.

Length	Total CDS	Percentage(%)
**0-300bp**	41,409	39.90
**300-500bp**	21,178	20.40
**500-1000bp**	22,027	21.22
**1000-2000bp**	15,029	14.48
**2000-3000bp**	3,038	2.93
**>3000bp**	1,109	1.07
**Total number**	103,790	-

### Analysis of differentially expressed unigenes

Unigene annotation provided information of expression and functional annotation for unigenes. Information of functional annotation gave functional annotation of unigenes for protein, COG and GO.A total of 137,363 unigenes (71.37%) were annotated (Table 3).

**Table 3:** Summary statistics of functional annotation for unigenes.

Annotated databases	No. of unigene hit	Percentage
**NR**^a^	102,616	53.32%
**NT**^b^	118,804	61.73%
**Swiss-Prot**	66,268	34.43%
**KEGG**^c^	64,544	33.54%
**COG**^d^	42,890	22.29%
**GO**^e^	62,273	32.36%
**Total**	137,363	71.37%

^**a**^ NCBI non-redundant protein database,

^**b**^ NCBI nucleotide sequence database,

^**c**^ Kyoto Encyclopedia of Genes and Genomes database,

^**d**^Clusters of Orthologous Groups database,

^e^Gene Ontology database.

**Fig 2:**
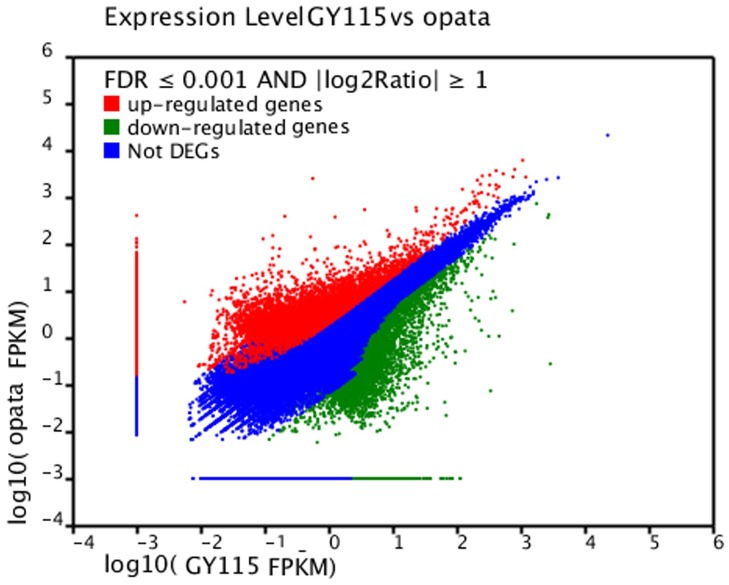
Differentially expressed genes between opata and Gy115. The genes were classified into three classes. Red genes are up-regulated if gene expression of right sample is larger than left sample. Green genes are down-regulated that gene expression of left sample is larger if right sample. Blue genes are not differentially expressed. The horizontal coordinates is the expression level of right and the vertical coordinates is the expression level of left sample.

To identify differentially expressed unigenes between Opata and Gy115, putative differentially expressed unigenes were identified on the basis of FPKM values calculated from the read counts mapped onto the reference transcriptome. A total of 23,642 unigenes were differentially expressed between Opata and Gy115 according to a comparison of expression levels with FDR ≤ 0.001 and |log2Ratio| ≥ 1 (Fig 2). Using Gy115 as a reference, 9945 up-regulated unigenes (with higher levels of expression in Opata) and 13,697 down-regulated unigenes (with higher levels of expression in Gy115) were identified. Significantly more unigenes were up-regulated in Opata than Gy115.

### GO functional classification of differentially expressed unigenes

**Fig 3:**
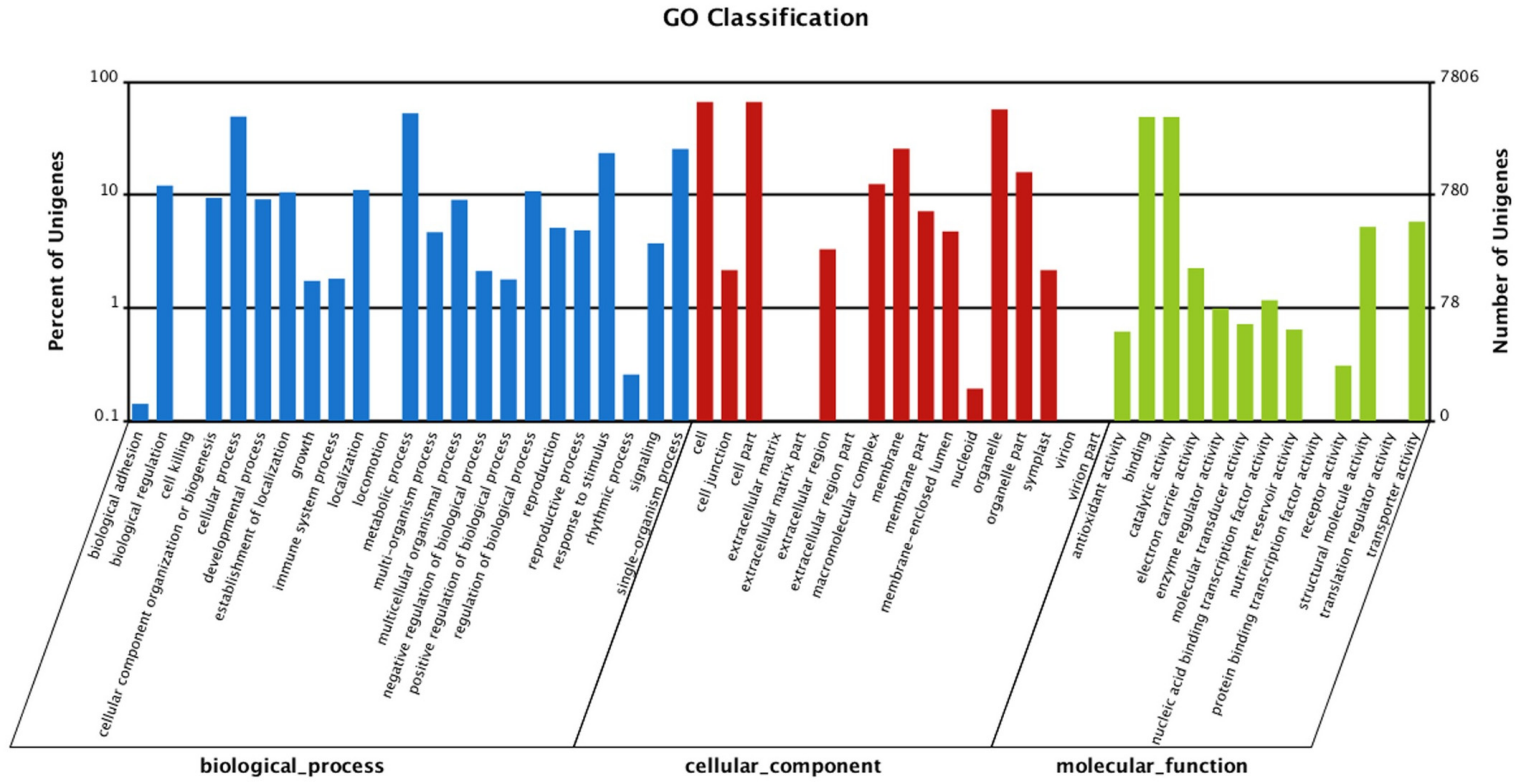
Gene Ontology (GO) classifications of differentially expressed unigenes. Unigenes were assigned to three categories: cellular components, molecular functions and biological process.

GO analysis assigned 16,833 differentially expressed unigenes to 53 subcategories. Among them, 5313 differentially expressed unigenes were involved in biological processes, 5467 were related to cellular components and 6053 were grouped under molecular functions. Within the biological process category, the great majority were related to ‘metabolic process’, ‘cellular process’ and ‘response to stimulus’. Within the cellular component category, the majority of differentially expressed unigenes were enriched in the subcategories ‘cell part’, ‘cell’ and ‘organelle’. Within the molecular function category, the largest proportion of differentially expressed unigenes were involved in ‘binding’ and ‘catalytic activity’–a relatively large number were related to ‘transporter activity’ (Fig 3), which may play an important role in the transport of ions, small molecules and macromolecules (e.g. amino acids and proteins).

### KEGG pathway mapping of differentially expressed unigenes

KEGG is the major public pathway-related database [24]. A total of 23,642 differentially expressed unigenes were annotated, and 9263 were mapped onto 127 pathways in the KEGG database. Most differentially expressed unigenes were involved in ‘Metabolic pathways’ (3055 unigenes), followed by ‘RNA transport’ (1379), ‘Biosynthesis of secondary metabolites’ (1248) and ‘mRNA surveillance pathway’ (1032) (S1 Table).

### Characteristics of unigenes related to flavonoid biosynthesis in the transcriptome of purple pericarp of Gy115

In the transcriptome of purple pericarp of Gy115, 143 unigenes related to flavone biosynthesis were identified. These unigenes were respectively grouped with16 structural genes: *ANS*, *bifunctional dihydroflavonol 4-reductase/flavanone 4-reductase* (*DFR*), *caffeoyl-CoAO-methyltransferase* (*CCoAOMT*), *CHI*, *CHS*, *coumaroylquinate* (*coumaroylshikimate*) *3ʹ-monooxygenase* (*C3ʹH*), *F3H*, *F3ʹ5ʹH*, *F3ʹH*, *flavonol synthase* (*FLS*), *leucoanthocyanidin reductase* (*LAR*), *leucoanthocyanidin dioxygenase* (*LDOX*), *shikimate O-hydroxycinnamoyltransferase* (*HCT*), *trans-cinnamate 4-monooxygenase* (*C4H*), *MYB* and *MYC*. The key genes for anthocyanin biosynthesis were *ANR*, *DFR*, *CHI*, *CHS*, *C3ʹH*, *F3ʹ5ʹH*, *F3ʹH*, *LDOX*, *LAR*, *F3H*, *MYB* and *MYC*, while *C3ʹH*, *HCT*, *C4H* and *FLS* competed for the same substrate in anthocyanin biosynthesis for producing other kind of flavonoid compounds. The maximum length of most unigenes related to flavonoid biosynthesis was greater than that of the reference genes. Because of the three copies of genome groups in common wheat, several unigenes were found to match one gene following blast searching in the unigene database. Some assembled unigenes were shorter than the reference genes. The average length of the assembled unigenes was greater than that of reference genes except for *CHS*, *C3ʹH*, *F3ʹ5ʹH*, *F3ʹH*, *HCT* and *C4H* (Table 4).

**Table 4:** The length of unigenes in Gy115 and reference genes relative to flavonoids biosynthesis.

Gene	KEGG ORTHOLOGY	KEGG ENZYME	Reference genes (bp)			Assemblied unigenes (bp)		
			maximum	minimum	Average	maximum	minimum	Average
**ANR**	K08695	1.3.1.77	1041	567	966	4765	156	1122
**DFR**	K13082	1.1.1.234 1.1.1.219	1170	1044	1084	4765	2768	3767
**CCoAOMT**	K00588	2.1.1.104	1014	609	782	3898	168	835
**CHI**	K01859	5.5.1.6	702	408	634	1138	1121	1130
**CHS**	K00660	2.3.1.74	1206	669	1169	1714	159	626
**C3'H**	K09754	1.14.13.36	1545	522	1436	2038	169	762
**F3'5'H**	K13083	1.14.13.88	1599	630	1211	1959	155	676
**F3'H**	K05280	1.14.13.21	1608	156	1381	3326	442	1109
**FLS**	K05278	1.14.11.23	1038	927	994	1841	158	1054
**LDOX**	K05277	1.14.11.19	1200	348	949	1642	1559	1610
**LAR**	K13081	1.17.1.3	1077	1071	1074	1356	1243	1305
**F3H**	K00475	1.14.11.9	1131	825	1088	1841	1554	1652
**HCT**	K13065	2.3.1.133	1422	1128	1321	1793	155	925
**C4H**	K00487	1.14.13.11	1620	954	1512	2087	163	972
**MYB**	K09422		738	738	738	1719	174	981
**MYC**	K13422		1707	1707	1707	5540	201	4085

**Fig 4:**
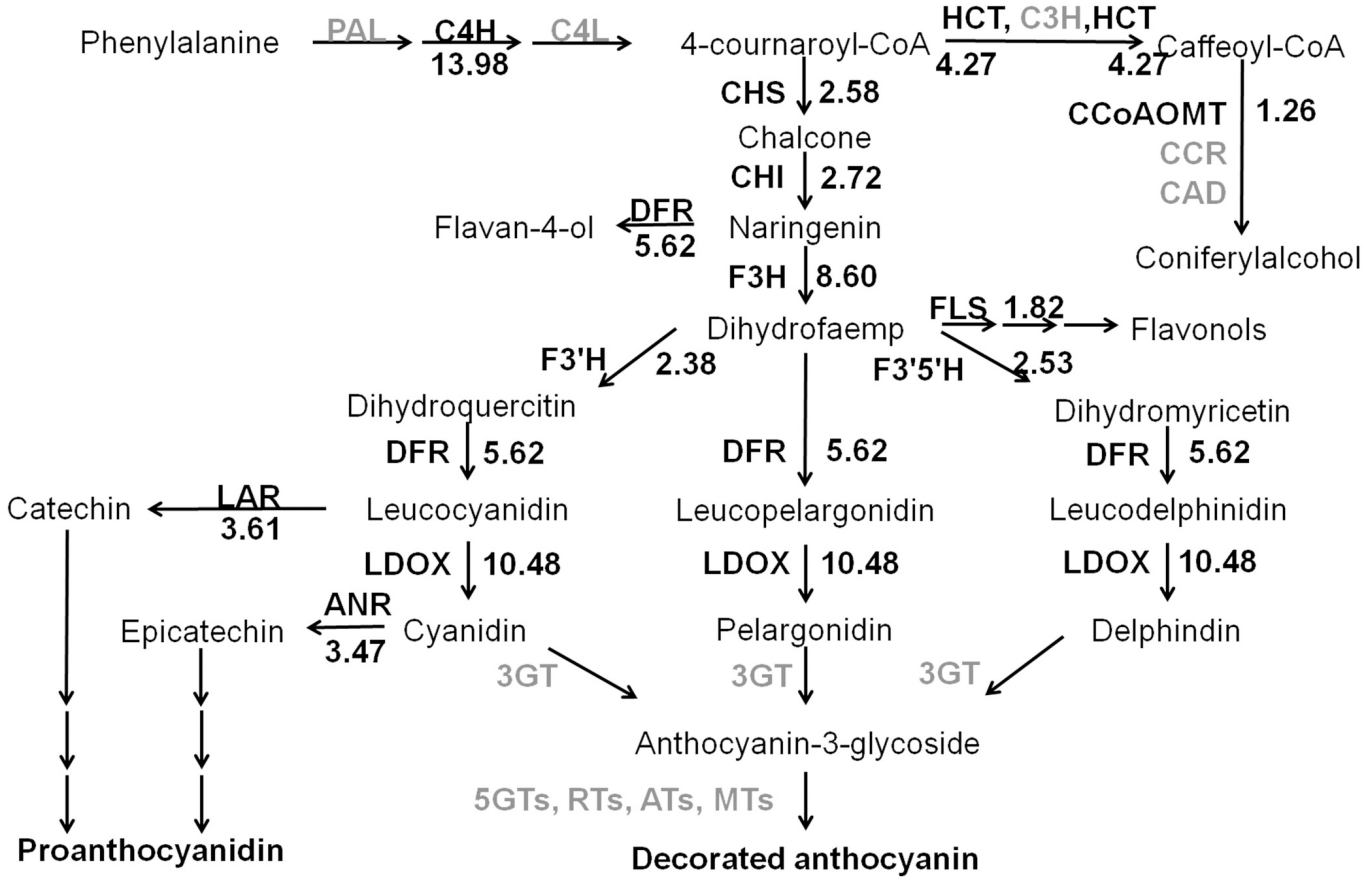
The expression differences of structural genes in the phenylalanine pathway. Arrow shows the metabolic stream, abbreviation left or upward arrows represent the genes catalyzing the progress, the light abbreviation means these genes were found in assembly unigenes, the number represent the in crease time of the expression in purple pericarp against white pericarp.

*PAT*, *C4L*, *3GT*, *5G*s, *RT*s, *AT*s and *MT*s, the key genes for anthocyanin biosynthesis, were found in the transcriptome of purple pericarp of Gy115 (Fig 4); and*3GT*, *5G*s, *RT*s, *AT*s and *MT*s were downstream of the pathway. The downstream genes should show more differentiation than genes in middle of the pathway, so it was hard to predict the sequence consistently. *PAT* and *C4L* were upstream of the anthocyanin biosynthesis pathway. The reasons for uncovering these genes is likely that they execute functions with relatively low expression.

### Expression profiles of genes related to anthocyanin biosynthesis in pericarps of white and purple grain

To compare relative expressions, the FPKM values of the same genes were accumulated, and their relative expression levels noted in the anthocyanin biosynthesis pathway. All genes were found to have more expression in purple compared with white pericarp, even those related to the biosynthesis of flavan-4-ol, coniferyl alcohol and proanthocyanidin. The anthocyanin biosynthesis pathway was more complete than other pathways, while only a few genes of other pathways were identified in this experiment. In the whole pathway, one copy of *C4H* had the maximum increase in expression of3085 times. This gene was the key gene deciding the metabolic stream of caffeine and other related compounds. The total expression level in Gy115 was 4.27 times that in Opata. Its completing gene *CHS* is related to anthocyanin biosynthesis, and had 2.58 times higher expression in Gy115 than in Opata—it was the only competing gene with higher expression than corresponding genes in the whole anthocyanin biosynthesis pathway. *DFR* could induce naringenin into flaval-4-ol, and increased by 5.62 times; while *F3H* inducing the same substrate naringenin into dihydrofaemp had an increase of 8.60 times. *FLS* directing dihydrofaemp into flavonols increased by1.82 times, and three competing genes *F3ʹH*, *F3ʹ5ʹH* and *DFR* were increased2.38, 2.53 and 5.62 times, respectively—all increased by more than *FLS* did. *LAR* increased by3.61 times, while *LDOX*by10.48 times in the side path from leucocyanidin to catechin and cyanidin. Overall, it could be speculated that the anthocyanin biosynthesis pathway was activated in purple pericarp, and the purple traits were the result of anthocyanin biosynthesis.

### Candidate genes for purple grain trait in wheat

Previous researchers found two dominant genes determining the purple grain trait, but the specific genes were not isolated. The purple grain trait of Gy115 came from tetraploid wheat, and it has been confirmed that the trait is controlled by homoallelic *Pp-1* genes and *Pp3*, on chromosomes 7BL and 2AL, respectively. The genes related to anthocyanin biosynthesis were assigned to the reference CDS with wheat chromosome location, and the genes residing on chromosomes 7BL and 2AL were chosen and analyzed. *MYB* and *CHS* genes were onchromosome7BL, and many genes were located on chromosome 2AL: *MYC*, *CHS*, *LDOX*, *F3ʹH*, *F3H*, *F3ʹ5ʹH*, *ANS* and *MYB*. Genes *CHS* and *MYB* were on chromosome 7BL, and both had three unigenes. One *MYB* unigene had lower expression in Gy115 compared with Opata but the other two unigenes showed higher expressionsof18.25 and 2.89 times. Three *CHS* unigenes had increased relative expression of3.63, 4.29 and 5.03 times. In chromosome 2AL, 52 unigenes were aligned in the anthocyanin biosynthesis pathway, and nine of these had higher expression in Opata than Gy115; so the nine unigenes could not be considered as candidate genes for purple grain. The expression increase for the remaining unigenes were in the range 2.23–3565. *CHS*, *LDOX*, *F3ʹH*, *F3H*, *F3ʹ5ʹH* and *ANS* are structural genes of anthocyanin biosynthesis, and make up the midstream of the pathway. *MYB* and *MYC* are regulatory genes, and could regulate the expression of structural genes, while the structural genes do not regulate expression of regulatory genes.

## Discussion

Most plants can accumulate anthocyanins in their tissues and organs. The structural and regulatory genes of anthocyanin biosynthesis should occupy an approprite proportion of expression relative to other genes in the kinds of cells that accumulate anthocyanins. Thus, the relative expression of anthocyanin biosynthesis genes should be relatively constant compared with all genes in these organs, meaning that the same content of RNA should contain an appreciable content of structural genes. In 8 Gb of data of RNA sequencing of purple pericarps of Gy115 full of anthocyanin, the longest of both regulatory and structural unigenes were longer than reference genes. All the longest unigenes encoded intact functional proteins. For example, the maximum of *MYB* was 1088bp, and contained the CDS of735bp. This means that the genes of anthocyanin biosynthesis could be isolated from the tissues and organs of plants with high anthocyanin content, and without genome information, based on RNA-Seq. Although the whole coding region of some genes can be obtained with current high-throughput sequencing, some unigenes could not encode the functional gene. Common wheat is hexaploid, with almost three copies of every gene compared with one in diploid plants, and expression of each copy in the total genome is diluted by the large genome. In diploid plants, single genes should be richer in cells accumulating anthocyanin and so it may be easier to isolate this kind of genes from diploid plants.

Within the biological process category, the great majority were related to ‘metabolic process’ in GO analysis. The pathways containing the most differentially expressed unigenes were involved in ‘Metabolic pathways’ in the KEGG database. The phenylpropanoid pathway, in which many compounds are biosynthesized, changed more obviously than other metabolic pathways. Compared with other compounds, the genes related to anthocyanin biosynthesis had higher expression levels in the purple pericarp of Gy115 than in Opata. Moreover, more structural genes were identified in the anthocyanin biosynthesis pathway in this RNA-Seq experiment. Thus, the whole metabolic pathway of anthocyanin biosynthesis was activated in the purple pericarp of Gy115. This confirmed that the purple grain trait was likely related to anthocyanin biosynthesis.

Previous research showed that the genetic basis of purple grain pigmentation resides in the action of the homoallelic *Pp-1* genes and *Pp3* [13,15,16,25,26]. The former map to the short arms of the homologous group 7 chromosomes [13,15,25,26] and the latter to chromosome arm 2AL [13,15,26]. In our transcriptome analysis of purple grain, 52 unigenes were related to anthocyanin biosynthesis, and had obvious expression differences in pericarps of Opata and Gy115. Nine of these unigenes had lower expression in Gy115, but 43 had higher expression. Adding five segments from chromosome 7BL, a total of 48 unigenes should be checked to determine the key genes for the purple grain trait. Fortunately, the *Pp-1* genes were confirmed as orthologs of both maize *C1* and rice *OsC1*, which encode MYB-like transcription factors (TFs) responsible for the activation of structural genes encoding various enzymes participating in anthocyanin synthesis [27,28]. Similarly, *Pp3* has been shown to be orthologous to both *Pb*/*Ra* in rice [28,29,30] and *Lc*/*R* in maize [31], which encode MYC-like TFs underlying the regulation of anthocyanin synthesis. A regulatory role of the *Pp* genes was also confirmed by functional analysis of the anthocyanin synthesis structural genes in wheat near-isogenic lines differing in the allelic state of the *Pp-1* and *Pp3* genes (both genes were in a dominant or recessive state) [32]. Recently, *TaMYC1* isolated from chromosome 2A was identified as a likely candidate for *Pp3* [33], but its function requires further confirmation. In our experiment, *MYC* and *MYB* were found onchromosomes2AL and 7BL, respectively. *MYB* and *MYC* are regulatory genes, and can induce expression of structural genes, while the structural genes cannot induce expression of regulatory genes. Thus, it should be useful to use *MYB* and *MYC* as candidate genes to explore the purple grain trait rather than structural genes, even though only *MYB* and *MYC* had many unigenes. *MYB* at chromosome 7BL had three unigenes, while *MYC* had four unigenes in the present study. Both *Pp-1* and *Pp3* are believed to be composed of only one gene. One possible reason for this situation is that these genes had several transcript copies; and the other reason is that the identities of genes on other chromosomes were confused because of high similarities in homologous genes in common wheat.

## Supporting Information

S1 Fig Electrophoresis of RNA of Gy115 and Opata. (DOCX)

S1 Table Differentially expressed unigenes with significantly enriched pathways. (DOC)
