# Supplementary figures and images for: Transcriptome Analysis of Purple Pericarps in Common Wheat (Triticum aestivum L.)
Source: PLoS One. 2016 May 12;11(5):e0155428. doi: 10.1371/journal.pone.0155428 (PMC4865117; doi:10.1371/journal.pone.0155428)

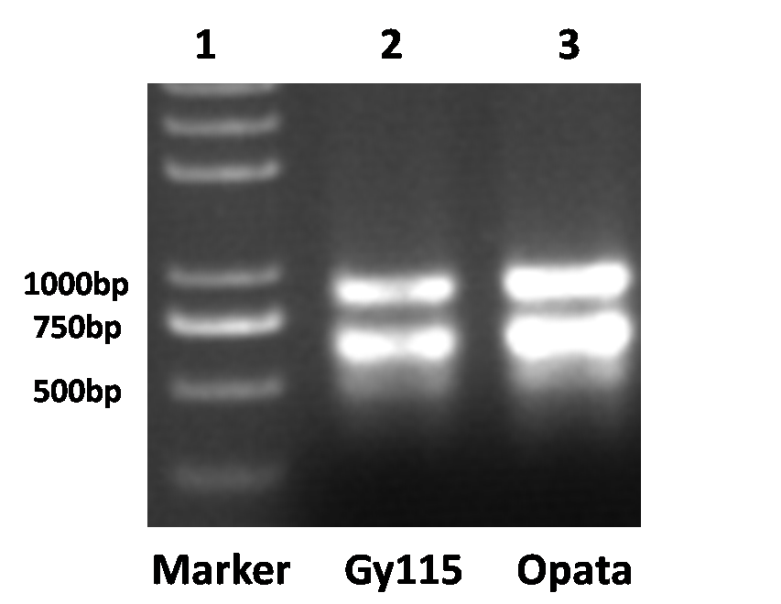


Supplemental Figure 1. Electrophoresis of RNA of Gy115 and Opata.

1. Marker 2. Gy115 3. Opata

Supplement: S1 Fig — (DOCX) [file pone.0155428.s001.docx]
