# Supplementary material for: Transcriptome Analysis of Purple Pericarps in Common Wheat (Triticum aestivum L.)
Source: PLoS One. 2016 May 12;11(5):e0155428. doi: 10.1371/journal.pone.0155428 (PMC4865117; doi:10.1371/journal.pone.0155428)
Supplement: S1 Table — (DOC) [file pone.0155428.s002.doc]

**Supplemental Table.1 Differentially expressed unigenes with significantly enriched pathways**

| **Number** | **Pathway** | **DEGs(9263)** | **All genes(64544)** | **P-value** |
| --- | --- | --- | --- | --- |
| 1 | Metabolic pathways | 3055 | 19566 (30.31%) | 1.09496E-09 |
| 2 | RNA transport | 1379 | 9930 (15.38%) | 0.9268727 |
| 3 | Biosynthesis of secondary metabolites | 1248 | 7116 (11.03%) | 1.23618E-15 |
| 4 | mRNA surveillance pathway | 1032 | 8197 (12.7%) | 0.9999996 |
| 5 | Endocytosis | 962 | 7013 (10.87%) | 0.9482218 |
| 6 | Glycerophospholipid metabolism | 947 | 6959 (10.78%) | 0.9712262 |
| 7 | Ether lipid metabolism | 895 | 6468 (10.02%) | 0.8969011 |
| 8 | Plant-pathogen interaction | 723 | 4233 (6.56%) | 1.67437E-07 |
| 9 | Ribosome | 537 | 1820 (2.82%) | 6.10047E-64 |
| 10 | Starch and sucrose metabolism | 449 | 2667 (4.13%) | 0.000138453 |
| 11 | Purine metabolism | 398 | 2325 (3.6%) | 8.44916E-05 |
| 12 | Pyrimidine metabolism | 396 | 2230 (3.46%) | 3.20015E-06 |
| 13 | Pentose and glucuronate interconversions | 350 | 2003 (3.1%) | 4.47744E-05 |
| 14 | Spliceosome | 350 | 3064 (4.75%) | 0.9999995 |
| 15 | RNA polymerase | 341 | 1761 (2.73%) | 3.12366E-09 |
| 16 | Plant hormone signal transduction | 288 | 2288 (3.54%) | 0.9940944 |
| 17 | Protein processing in endoplasmic reticulum | 245 | 1746 (2.71%) | 0.660585 |
| 18 | Glycolysis / Gluconeogenesis | 227 | 1257 (1.95%) | 0.000137765 |
| 19 | Phenylpropanoid biosynthesis | 224 | 1151 (1.78%) | 1.01821E-06 |
| 20 | RNA degradation | 218 | 1619 (2.51%) | 0.8570991 |
| 21 | Oxidative phosphorylation | 211 | 957 (1.48%) | 7.62806E-11 |
| 22 | Ribosome biogenesis in eukaryotes | 204 | 1444 (2.24%) | 0.6085613 |
| 23 | Galactose metabolism | 149 | 842 (1.3%) | 0.003794849 |
| 24 | Amino sugar and nucleotide sugar metabolism | 147 | 766 (1.19%) | 0.000130441 |
| 25 | Fructose and mannose metabolism | 147 | 774 (1.2%) | 0.000212308 |
| 26 | Homologous recombination | 135 | 560 (0.87%) | 5.5173E-10 |
| 27 | Flavonoid biosynthesis | 132 | 546 (0.85%) | 7.0088E-10 |
| 28 | Pentose phosphate pathway | 122 | 701 (1.09%) | 0.01333292 |
| 29 | Pyruvate metabolism | 115 | 645 (1%) | 0.007863986 |
| 30 | Stilbenoid, diarylheptanoid and gingerol biosynthesis | 108 | 449 (0.7%) | 3.08742E-08 |
| 31 | Phagosome | 107 | 604 (0.94%) | 0.01198159 |
| 32 | Peroxisome | 104 | 500 (0.77%) | 5.42801E-05 |
| 33 | Biosynthesis of unsaturated fatty acids | 101 | 317 (0.49%) | 1.80757E-15 |
| 34 | Cysteine and methionine metabolism | 96 | 654 (1.01%) | 0.4220727 |
| 35 | ABC transporters | 91 | 658 (1.02%) | 0.6660081 |
| 36 | Ubiquitin mediated proteolysis | 91 | 913 (1.41%) | 0.9999723 |
| 37 | Glyoxylate and dicarboxylate metabolism | 90 | 423 (0.66%) | 7.13079E-05 |
| 38 | Cyanoamino acid metabolism | 89 | 480 (0.74%) | 0.006424334 |
| 39 | Fatty acid metabolism | 87 | 372 (0.58%) | 2.17268E-06 |
| 40 | Tryptophan metabolism | 86 | 353 (0.55%) | 4.1168E-07 |
| 41 | Nucleotide excision repair | 82 | 569 (0.88%) | 0.5020513 |
| 42 | Zeatin biosynthesis | 81 | 445 (0.69%) | 0.01392236 |
| 43 | Phenylalanine metabolism | 78 | 437 (0.68%) | 0.02386813 |
| 44 | Glutathione metabolism | 76 | 548 (0.85%) | 0.6452945 |
| 45 | Cutin, suberine and wax biosynthesis | 75 | 304 (0.47%) | 1.30957E-06 |
| 46 | Limonene and pinene degradation | 75 | 355 (0.55%) | 0.000335321 |
| 47 | Citrate cycle (TCA cycle) | 73 | 437 (0.68%) | 0.09207425 |
| 48 | Arginine and proline metabolism | 72 | 551 (0.85%) | 0.821917 |
| 49 | Tyrosine metabolism | 70 | 357 (0.55%) | 0.003864619 |
| 50 | Carbon fixation in photosynthetic organisms | 69 | 375 (0.58%) | 0.01733131 |
| 51 | Fatty acid biosynthesis | 65 | 203 (0.31%) | 1.31792E-10 |
| 52 | Glycine, serine and threonine metabolism | 62 | 429 (0.66%) | 0.4972546 |
| 53 | Carotenoid biosynthesis | 61 | 320 (0.5%) | 0.01198329 |
| 54 | Diterpenoid biosynthesis | 60 | 269 (0.42%) | 0.000292299 |
| 55 | Glycerolipid metabolism | 60 | 369 (0.57%) | 0.1646243 |
| 56 | Ascorbate and aldarate metabolism | 58 | 294 (0.46%) | 0.006951396 |
| 57 | Valine, leucine and isoleucine degradation | 58 | 350 (0.54%) | 0.1339417 |
| 58 | Flavone and flavonol biosynthesis | 57 | 221 (0.34%) | 5.51633E-06 |
| 59 | Terpenoid backbone biosynthesis | 56 | 380 (0.59%) | 0.4370711 |
| 60 | Propanoate metabolism | 54 | 323 (0.5%) | 0.1287696 |
| 61 | Benzoxazinoid biosynthesis | 53 | 158 (0.24%) | 9.94879E-10 |
| 62 | Isoflavonoid biosynthesis | 53 | 215 (0.33%) | 4.3874E-05 |
| 63 | Proteasome | 53 | 315 (0.49%) | 0.1212459 |
| 64 | Alanine, aspartate and glutamate metabolism | 52 | 361 (0.56%) | 0.5114701 |
| 65 | DNA replication | 49 | 293 (0.45%) | 0.1412046 |
| 66 | Basal transcription factors | 49 | 409 (0.63%) | 0.9282553 |
| 67 | Butanoate metabolism | 46 | 220 (0.34%) | 0.00514418 |
| 68 | Nitrogen metabolism | 44 | 297 (0.46%) | 0.4346939 |
| 69 | Mismatch repair | 43 | 256 (0.4%) | 0.1518725 |
| 70 | Circadian rhythm - plant | 43 | 279 (0.43%) | 0.3309304 |
| 71 | Phosphatidylinositol signaling system | 43 | 335 (0.52%) | 0.807077 |
| 72 | Other glycan degradation | 42 | 284 (0.44%) | 0.4421642 |
| 73 | beta-Alanine metabolism | 41 | 243 (0.38%) | 0.1513224 |
| 74 | Lysine degradation | 41 | 304 (0.47%) | 0.6909421 |
| 75 | Inositol phosphate metabolism | 38 | 278 (0.43%) | 0.6531645 |
| 76 | Base excision repair | 37 | 235 (0.36%) | 0.2969927 |
| 77 | alpha-Linolenic acid metabolism | 37 | 266 (0.41%) | 0.6081018 |
| 78 | Photosynthesis - antenna proteins | 35 | 65 (0.1%) | 9.9649E-14 |
| 79 | Valine, leucine and isoleucine biosynthesis | 35 | 221 (0.34%) | 0.2908198 |
| 80 | Steroid biosynthesis | 35 | 251 (0.39%) | 0.600497 |
| 81 | Protein export | 34 | 279 (0.43%) | 0.8698092 |
| 82 | Photosynthesis | 33 | 171 (0.26%) | 0.04512529 |
| 83 | Aminoacyl-tRNA biosynthesis | 31 | 444 (0.69%) | 0.9999996 |
| 84 | Porphyrin and chlorophyll metabolism | 29 | 275 (0.43%) | 0.9745059 |
| 85 | Tropane, piperidine and pyridine alkaloid biosynthesis | 28 | 137 (0.21%) | 0.03224748 |
| 86 | Selenocompound metabolism | 27 | 151 (0.23%) | 0.1319986 |
| 87 | SNARE interactions in vesicular transport | 27 | 172 (0.27%) | 0.3383759 |
| 88 | Sphingolipid metabolism | 25 | 174 (0.27%) | 0.5304957 |
| 89 | Regulation of autophagy | 25 | 232 (0.36%) | 0.9549418 |
| 90 | Linoleic acid metabolism | 23 | 114 (0.18%) | 0.05508075 |
| 91 | Histidine metabolism | 22 | 132 (0.2%) | 0.2569745 |
| 92 | Isoquinoline alkaloid biosynthesis | 22 | 146 (0.23%) | 0.4377273 |
| 93 | Phenylalanine, tyrosine and tryptophan biosynthesis | 22 | 227 (0.35%) | 0.9858681 |
| 94 | N-Glycan biosynthesis | 22 | 246 (0.38%) | 0.996067 |
| 95 | Ubiquinone and other terpenoid-quinone biosynthesis | 21 | 155 (0.24%) | 0.6468655 |
| 96 | Fatty acid elongation | 21 | 163 (0.25%) | 0.7361052 |
| 97 | Lysine biosynthesis | 19 | 102 (0.16%) | 0.1384212 |
| 98 | Natural killer cell mediated cytotoxicity | 19 | 153 (0.24%) | 0.7847684 |
| 99 | Pantothenate and CoA biosynthesis | 18 | 125 (0.19%) | 0.5326486 |
| 100 | Sulfur metabolism | 15 | 117 (0.18%) | 0.7204802 |
| 101 | Circadian rhythm - mammal | 14 | 83 (0.13%) | 0.2997048 |
| 102 | Glycosylphosphatidylinositol(GPI)-anchor biosynthesis | 13 | 199 (0.31%) | 0.9998227 |
| 103 | Taurine and hypotaurine metabolism | 12 | 68 (0.11%) | 0.264825 |
| 104 | Brassinosteroid biosynthesis | 12 | 88 (0.14%) | 0.6220013 |
| 105 | Synthesis and degradation of ketone bodies | 11 | 46 (0.07%) | 0.05735084 |
| 106 | Glucosinolate biosynthesis | 11 | 79 (0.12%) | 0.5919509 |
| 107 | Glycosaminoglycan degradation | 11 | 122 (0.19%) | 0.971336 |
| 108 | Arachidonic acid metabolism | 10 | 81 (0.13%) | 0.7429478 |
| 109 | Anthocyanin biosynthesis | 9 | 29 (0.04%) | 0.01705303 |
| 110 | Riboflavin metabolism | 9 | 91 (0.14%) | 0.9197881 |
| 111 | One carbon pool by folate | 9 | 98 (0.15%) | 0.9529207 |
| 112 | C5-Branched dibasic acid metabolism | 7 | 29 (0.04%) | 0.11166 |
| 113 | Monoterpenoid biosynthesis | 7 | 34 (0.05%) | 0.207253 |
| 114 | Sesquiterpenoid and triterpenoid biosynthesis | 7 | 47 (0.07%) | 0.5206307 |
| 115 | Folate biosynthesis | 7 | 65 (0.1%) | 0.8422357 |
| 116 | Vitamin B6 metabolism | 7 | 74 (0.11%) | 0.921043 |
| 117 | Other types of O-glycan biosynthesis | 6 | 46 (0.07%) | 0.6633938 |
| 118 | Nicotinate and nicotinamide metabolism | 6 | 52 (0.08%) | 0.775589 |
| 119 | Glycosphingolipid biosynthesis - ganglio series | 6 | 71 (0.11%) | 0.9526586 |
| 120 | Indole alkaloid biosynthesis | 5 | 42 (0.07%) | 0.739511 |
| 121 | Glycosphingolipid biosynthesis - globo series | 5 | 59 (0.09%) | 0.9388683 |
| 122 | Non-homologous end-joining | 5 | 61 (0.09%) | 0.9494546 |
| 123 | Thiamine metabolism | 4 | 40 (0.06%) | 0.845166 |
| 124 | Betalain biosynthesis | 3 | 15 (0.02%) | 0.3673918 |
| 125 | Sulfur relay system | 3 | 51 (0.08%) | 0.9832331 |
| 126 | Lipoic acid metabolism | 1 | 9 (0.01%) | 0.752007 |
| 127 | Biotin metabolism | 1 | 12 (0.02%) | 0.8442006 |
